# Inactivation and Recovery of High Quality RNA From Positive SARS-CoV-2 Rapid Antigen Tests Suitable for Whole Virus Genome Sequencing

**DOI:** 10.3389/fpubh.2022.863862

**Published:** 2022-05-03

**Authors:** Guerrino Macori, Tristan Russell, Gerald Barry, Siobhán C. McCarthy, Leonard Koolman, Patrick Wall, Donal Sammin, Grace Mulcahy, Séamus Fanning

**Affiliations:** ^1^Centre for Food Safety, School of Public Health, Physiotherapy and Sports Science, University College Dublin, Dublin, Ireland; ^2^School of Veterinary Medicine, University College Dublin, Dublin, Ireland; ^3^Department of Agriculture, Food and the Marine Laboratories, Celbridge, Ireland; ^4^Conway Institute, University College Dublin, Dublin, Ireland

**Keywords:** antigen testing, RT-qPCR, whole virus genome sequencing, lateral flow device, rapid antigen diagnostic test

## Abstract

The diagnostic protocol currently used globally to identify Severe Acute Respiratory Syndrome Coronavirus 2 (SARS-CoV-2) infection is RT-qPCR. The spread of these infections and the epidemiological imperative to describe variation across the virus genome have highlighted the importance of sequencing. SARS-CoV-2 rapid antigen diagnostic tests (RADTs) are designed to detect viral nucleocapsid protein with positive results suggestive of the presence of replicating virus and potential infectivity. In this study, we developed a protocol for recovering SARS-CoV-2 RNA from “spent” RADT devices of sufficient quality that can be used directly for whole virus genome sequencing. The experimental protocol included the spiking of RADTs at different concentrations with viable SARS-CoV-2 variant Alpha (lineage B.1.1.7), lysis for direct use or storage. The lysed suspensions were used for RNA extraction and RT-qPCR. In parallel, we also tested the stability of the viral RNA in the RADTs and the RNA extracted from the RADTs was used as a template for tiling-PCR and whole virus genome sequencing. RNA recovered from RADTs spiked with SARS-CoV-2 was detected through RT-qPCR with C_t_ values suitable for sequencing and the recovery from RADTs was confirmed after 7 days of storage at both 4 and 20°C. The genomic sequences obtained at each time-point aligned to the strain used for the spiking, demonstrating that sufficient SARS-CoV-2 viral genome can be readily recovered from positive-RADT devices in which the virus has been safely inactivated and genomically conserved. This protocol was applied to obtain whole virus genome sequence from RADTs ran in the field where the omicron variant was detected. The study demonstrated that viral particles of SARS-CoV-2 suitable for whole virus genome sequencing can be recovered from positive spent RADTs, extending their diagnostic utility, as a risk management tool and for epidemiology studies. In large deployment of the RADTs, positive devices could be safely stored and used as a template for sequencing allowing the rapid identification of circulating variants and to trace the source and spread of outbreaks within communities and guaranteeing public health.

## Introduction

The diagnostic protocol currently used globally to identify Severe Acute Respiratory Syndrome Coronavirus 2 (SARS-CoV-2) infection is RT-qPCR (1). RNA extracted from nasopharyngeal swabs is amplified to detect several viral structural and accessory genetic elements as suitable targets for this method (2). Although RT-qPCR has excellent sensitivity, the rapid spread of these infections and the epidemiological imperative to describe variation across the virus genome highlights the importance of sequencing (3). This in turn can enable refinement of detection methods (4) to facilitate the tracking of transmission pathways in nosocomial outbreaks (5) whilst highlighting superinfections and intra-host mutations resulting in the emergence of variants of concern (VOC) (6). SARS-CoV-2 rapid antigen detection tests (RADT) are designed to detect viral nucleocapsid protein with positive results suggestive of the presence of replicating virus and potential infectivity. RADT do not detect viral particle numbers as low as those detected by PCR, but are effective in detecting levels of virus likely to transmit infection (7). The frequent use of RADT testing in particular settings, such as meat processing plants (MPPs), can support risk-mitigation, in identifying and excluding highly infectious individuals from the workplace (8). The ability to recover viral RNA from spent positive RADT devices for subsequent whole virus genome sequencing (WvGS) would enable both the identification of virus lineage and definition of nucleotide polymorphisms, thus facilitating molecular epidemiological mapping of viral spread within these communities, as well as detecting the emergence of any new SARS-CoV-2 VOCs. This study provides proof of concept of using spent positive RADT kits to generate viral sequence data of sufficient quality to identify circulating variants and to trace the source and spread of outbreaks within communities.

## Methods

### Recovery of SARS CoV-2 RNA From “Spent” RADT Test Devices

In this study, we used the Abbott Panbio™ COVID-19 Ag Rapid Test Device kit (Nasal) (Abbott Laboratories Ltd., USA) as RADT spiked with viable SARS-CoV-2 variant Alpha, lineage B.1.1.7 (Human nCoV19 isolate/England/MIG457/2020) grown in Vero E6 cells with a titer of 1.8 × 10^4^ plaque forming units (PFU)/mL (9). For studying the correlation between recovery of the RNA from RADTs and concentration of SARS-CoV-2, the RADTs were inoculated in a 90° angle to the specimen well with 120 μL 1:500, 1:1,000, 1:2,000, 1:4,000, 1:8,000, and 1:16,000 dilutions of SARS-CoV-2 in duplicate. The buffer provided in the RADTs was used for preparing the dilutions. After inoculation, the RADTs were maintained on a flat surface for 15 min at room temperature, in accordance with the manufacturers' instructions. The appearance of control and test lines showed that the test was valid and capable of detecting cultured virus. The spent RADTs were then slowly filled with 700 μL viral lysis buffer (AVL) (QIAamp® Viral RNA Mini kit, Qiagen Ltd, UK) and then incubated for 10 min at room temperature. Each device was then transferred into a sterile 30 mL sample tube, vortexed for 5 s and centrifuged at 5,000 × *g* for 1 min. The lysed suspension (~700 μL) was then used as a template for RNA extraction using a programmable QIAcube Platform (Qiagen Ltd., UK) according to the manufacturer's instructions.

The same protocol was used for the preparation of RADTs inoculated with neat and 1:16,000 dilution of SARS-CoV-2 with the aim of testing the stability of the RNA in these devices following incubation for maximum 7 days at 4 and 20°C after addition of buffer AVL.

In order to validate virus inactivation, eluate recovered from RADTs spiked with 120 μL of neat SARS-CoV-2 and 700 μL Buffer AVL was added to Vero E6 cells. Before addition to Vero E6 cells, cytotoxic components of the AVL buffer were eliminated from eluate using detergent removal spin columns (ThermoFisher, UK), which were shown to recover 100% of virus (10). The protocol demonstrated that viable virus could no longer be detected in the eluate from positive RADT test devices to which AVL buffer was added, but viable virus was detected when 700 μL of PBS was added. RNA suitable for WvGS was recoverable.

### Protocol for Safe Virus Inactivation and Use for RT-qPCR and WvGS

**Figure 1:**
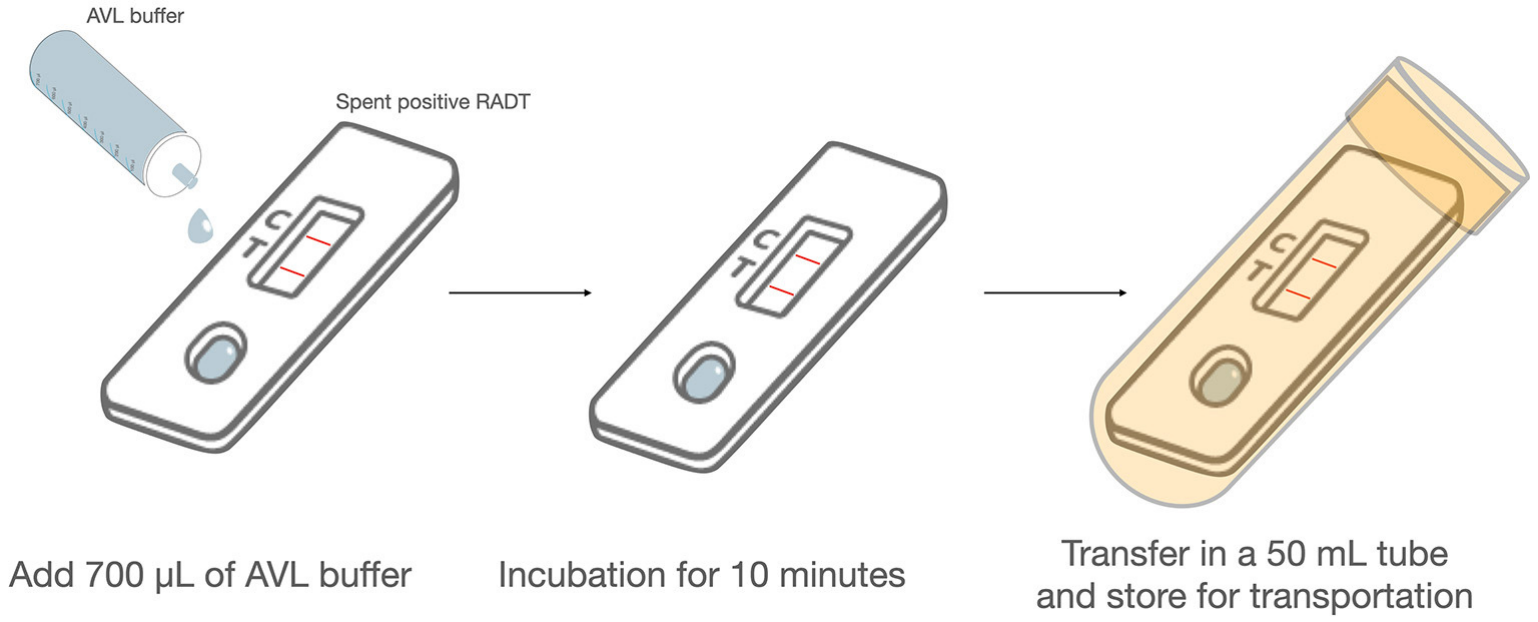
Protocol for safe virus inactivation of RADT on-site. The experimental protocol was modified to facilitate the safe handling of real-field positive RADTs. SARS-CoV-2 positive RADTs are inoculated on-site with 700 μL AVL provided in ready to use aliquots and transferred gently drop-by-drop. After an incubation of 10 min, the RADT is transferred into a 50 mL sample tube, sealed and maintained at 4 or 20°C, then delivered to the laboratory.

The protocol described above was modified to facilitate the safe handling of real-field positive RADTs. SARS-CoV-2 positive RADTs were inoculated on-site with 700 μL AVL provided in ready to use aliquots in 1.5 mL tubes and transferred gently drop-by-drop using a single-use polyethylene Pasteur pipette (Fisher Scientific Ireland). After an incubation of 10 min, the RADT were transferred into a 50 mL sample tube, sealed and maintained at 4 or 20°C, then delivered to the laboratory (Figure 1). The next steps were performed in containment biosafety laboratory category 2 (BSL-2) facilities using standard BSL-2 work practices. The tubes were centrifuged for 5,000 × g for 1 min, and the discharged liquid collected in the bottom of the tubes (about 700 μL) was retrieved for RNA extraction and RT-qPCR using the method as described above. This protocol was used for the extraction of 30 RADTs from a number of MPPs in Ireland returning positive results. The samples were randomly chosen from a larger dataset of positive RADTs, including Abbott Panbio™ (Abbott Laboratories Ltd., USA) and Clinitest® Rapid COVID-19 Antigen Test (Siemens Healthineers, Germany) where voluntary participants from a number of MPPs provided their informed consent. Workers were invited to participate and provided with an information leaflet and consent form for signature. Anonymised data from the survey was provided to the research team, with ethical approval from UCD Human Research Ethics Committee (No.: LS-E-20-196-Mulcahy).

### RT-qPCR Detection of SARS-CoV-2 and Whole Viral Genome Sequencing

The presence of SARS-CoV-2 RNA in purified samples, either from the experimental protocols or positive RADTs from MPPs, was confirmed by RT-qPCR targeting the nucleocapsid genes 1 (N1) and 2 (N2) and the human RNase P (RP). Three single reactions were prepared using combined Primer/Probe Mix (1.5 μL) (IDT, USA) for each target (Table 1) adding DNase & RNase free water (6.5 μL) (Fisher Scientific Ireland), qScript XLT One-Step RT-qPCR ToughMix (2X) Low ROX (10 μL) (Quantabio, USA) and 2 μL of the template RNA, obtaining 20 μL of total reaction. A standard curve with 1:10 serial dilution of single stranded RNA (ssRNA) fragments of SARS-CoV-2 reference material (EU-JRC, Italy) was included on each RT-qPCR run along with negative extraction control. The cycling protocol of the complete reaction mix was incubated in a QuantStudio 5 Real-Time PCR System (Applied Biosystems, USA) as follows: cDNA Synthesis (50°C for 10 min), initial denaturation (95°C, for 1 min) PCR cycling (40 cycles) included denaturation (95°C for 10 s) extension and data collection step (60°C for 1 min).

**Table 1:** Panel of primer and probes used for the RT-qPCR used in this study.

**Label name**	**Description**	**Oligonucleotide sequence (5^′^> 3^′^)**	**Label**	**Final conc**.
2019- nCoV_N1-F	2019-nCoV_N1 Forward Primer	GAC CCC AAA ATC AGC GAA AT	None	500 nM
2019- nCoV_N1-R	2019-nCoV_N1 Reverse Primer	TCT GGT TAC TGC CAG TTG AAT CTG	None	500 nM
2019- nCoV_N1-P	2019-nCoV_N1 Probe	FAM-ACC CCG CAT TAC GTT TGG TGG ACC-BHQ1	FAM, BHQ-1	125 nM
2019- nCoV_N2-F	2019-nCoV_N2 Forward Primer	TTA CAA ACA TTG GCC GCA AA	None	500 nM
2019- nCoV_N2-R	2019-nCoV_N2 Reverse Primer	GCG CGA CAT TCC GAA GAA	None	500 nM
2019- nCoV_N2-P	2019-nCoV_N2 Probe	FAM-ACA ATT TGC CCC CAG CGC TTC AG-BHQ1	FAM, BHQ-1	125 nM
RP-F	RNase P Forward Primer	AGA TTT GGA CCT GCG AGC G	None	500 nM
RP-R	RNase P Reverse Primer	GAG CGG CTG TCT CCA CAA GT	None	500 nM
RP-P	RNase P Probe	FAM – TTC TGA CCT GAA GGC TCT GCG CG – BHQ-1	FAM, BHQ-1	125 nM

The extracted samples were also used as a template for multiplex PCR (tiling-PCR) according to the ARTIC panel (version 3) (11) and amplicons were prepared for WvGS, following the protocol for Illumina MiSeq sequencing (5). Raw data were quality assessed using FastQC (version 0.11.7) and pre-processed with fastp (version 0.20.1) (12). Consensus sequences were generated using the computational package iVar (version 1.0) (13). For phylogenetic analysis, sequences were aligned using a pipeline used previously (5, 6) which included the analysis with Nextclade (14) to identify differences between sequences and report sequence quality, while the pangolin tool was used for the assignment of epidemiological lineages (15). The sequences obtained were aligned using MAFFT (version 7) and for outlining the phylogenetic relationship among the sequences, a tree was generated with the Neighbor-Joining method (16) and visualized using FigTree (version 1.4.4) (https://github.com/rambaut/figtree).

## Results

### RT-qPCR Detection of SARS-CoV-2 in Positive Spent RADT Test Device and Genomic Comparison Experimental Study

**Figure 2:**
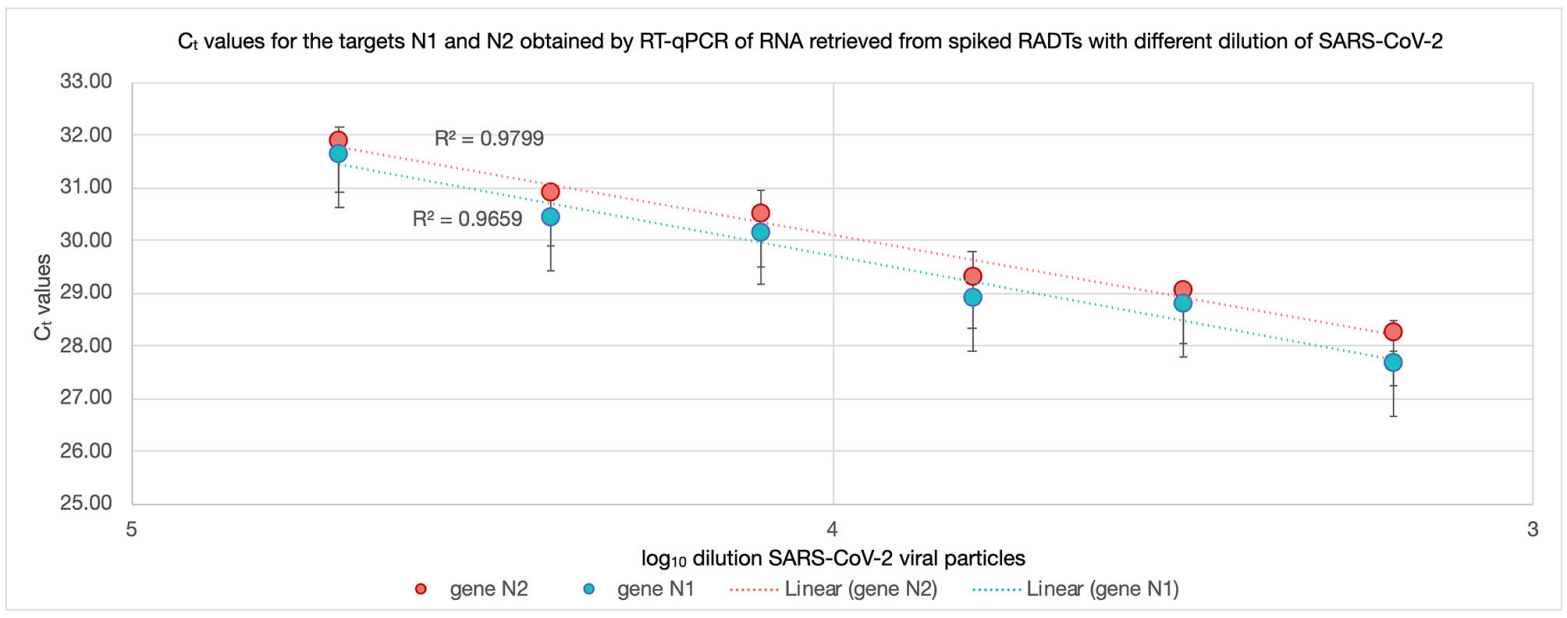
C_t_ values for N1 and N2 of RNA recovered from RADTs spiked with different concentrations of SARS-CoV-2 viral particles. The RNA recovered from RADTs spiked with SARS-CoV-2 was detected through RT-qPCR and the C_t_ values ranged between 27.49 and 31.80 for the gene N1 (blue) and 28.19 and 31.91 for the gene N2 (red) reported in the y-axes. The log_10_ of the SARS-CoV-2 viral particles dilutions is presented in the x-axes. The R-squared (*R*^2^) values are displayed in the dotted trend lines and the vertical bars on the points represent the standard deviation considering the average of the two C_t_ values recorded for each sample.

**Figure 3:**
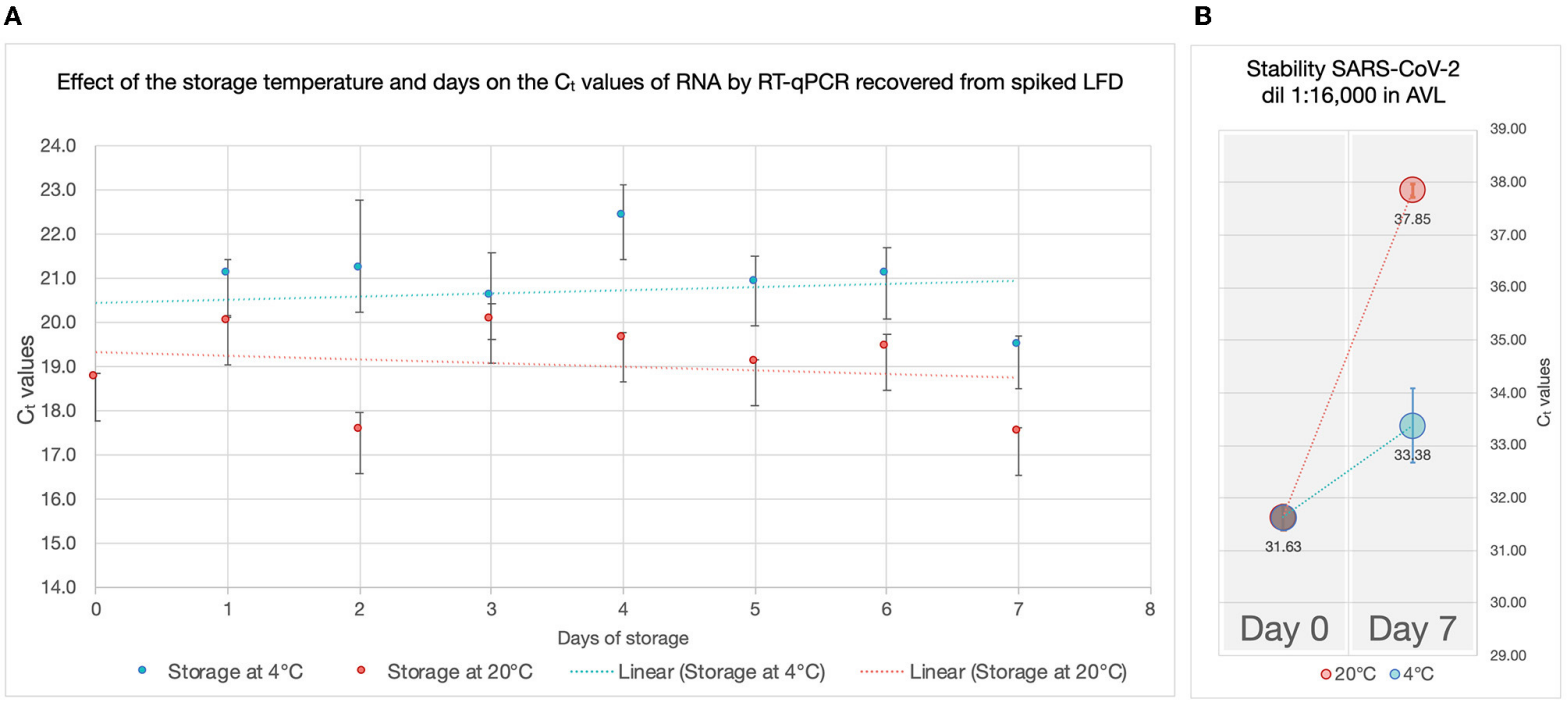
Effect of the storage temperature and time on the C_t_ values of RNA by RT-qPCR recovered from spiked LFD. **(A)** C_t_ values (y-axes) of the RNA recovered from RADTs spiked with a high titer of SARS-CoV-2 (1 × 10^3^ PFU/mL) following storage of RADTs at 4°C (blue) or 20°C (red) for 7 days. The vertical bars on the points represent the standard deviation considering the average of the two C_t_ values recorded for each sample and the trendlines (dotted lines) are shown. **(B)** Stability test. A diluted concentration (1:16,000) of the virus was used for spiking RADTs and stored at 4 and 20°C. The C_t_ values (y-axes) obtained on day 0 and 7 are shown in the plot along with the vertical bars on the points representing the standard deviation considering the average of the two C_t_ values recorded for the measurements.

**Figure 4:**
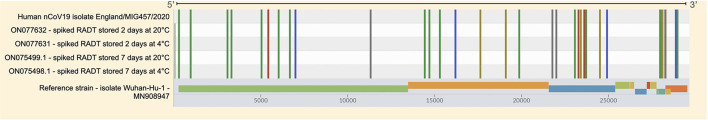
Sequence alignment of WvG sequences obtained from RNA during the stability test. The spiked RADTs were stored at 4°C and 20°C after the addition of buffer AVL and the extraction protocol was performed after 2 and 7 days. The GenBank id of the four samples are included in the left side of the figure, the Human nCoV19 isolate/England/MIG457/2020 used for the spiking is included. The colored lines represent the mutations and polymorphisms common to the four WvGs compared to the reference WvG (MN908947 - Severe acute respiratory syndrome coronavirus 2 isolate Wuhan-Hu-1).

All RADT spiked with SARS-CoV-2 produced a test and control line within 15 min of inoculation. The RNA recovered from RADTs spiked with SARS-CoV-2 was detected through RT-qPCR and the C_t_ values ranged between 27.49 and 31.80 for the gene N1 and 28.19 and 31.91 for the gene N2 (Figure 2) with ~1-C_t_ difference between each 1:2 dilution. There was no significant change in RNA detected by RT-qPCR overtime when RADTs were spiked with a high titer of SARS-CoV-2 (1 × 10^3^ PFU/mL) following storage of RADTs at 4 or 20°C (Figure 3A) using neat concentration of cultured SARS-CoV-2, while the stability study demonstrated reduced detection of viral RNA by RT-qPCR when RADTs were inoculated with the 1:16,000 dilution (C_t_ = 31.63) at day 7. Low amounts of RNA in RADTs appear to be more stable when incubated at 4°C (C_t_ = 33.38) compared to 20°C (C_t_ = 37.85) (Figure 3B). A total of four RNA samples extracted from RADTs from the stability test were sequenced, including two RADTs stored at 4°C and two at 20°C after addition of buffer AVL and extracted after 2 and 7 days. The alignment of the sequences showed the perfect alignment with the sequence of the Human SARS-CoV-2 variant Alpha (lineage B.1.1.7, isolate England/MIG457/2020) (17, 18) used for spiking the RADTs (Figure 4).

### RT-qPCR Detection of SARS-CoV-2 in Positive Spent RADT Test Device and Genomic Comparison in Field Samples

**Figure 5:**
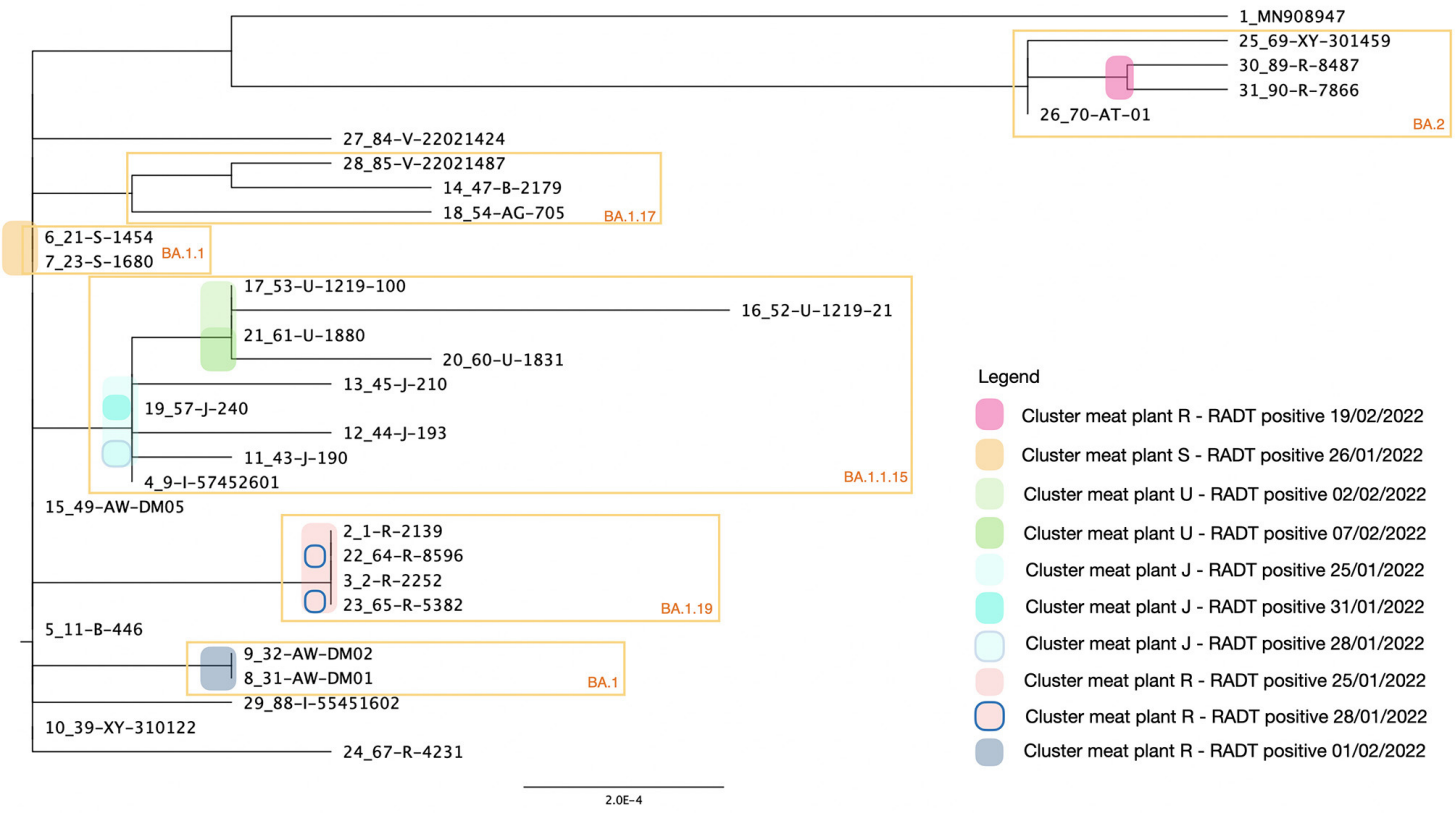
Maximum likelihood phylogenetic tree of the 30 field-samples. The sequences were obtained from the positive RADTs provided by operators in the MPPs and treated with the developed protocol described in this study. The six yellow squares highlight the clusters and the colored spots on the branches of the tree, group the related samples with the same date of isolation. The clusters are annotated based on the average distance among the sequences and the tree is rooted with the reference MN908947 (Severe acute respiratory syndrome coronavirus 2 isolate Wuhan-Hu-1).

The RNA extracted from positive RADTs from meat plants were tested for the presence of RNA of SARS-CoV-2 with the RT-qPCR. All the samples resulted positive with a Ct value included between 18.39 and 34.67 (Supplementary Table 1). All the samples were sequenced and the clade 21K (Omicron) was identified for 26 samples and four resulted 21L (Omicron). According to Nextclade Pango nomenclature, were identified seven different lineages: 9 BA.1.1.15 (30.0 %), 5 BA.1 (16.7 %), 4 BA.1.19 (13.3 %), 4 BA.1.1 (13.3 %), 4 BA (13.3 %), 3 BA.1.17 (10.0 %), and 1 BA.1.10 (3.3%). In addition the genome coverage ranged between 67.4 and 98.8% (Supplementary Table 1) while other parameters and details on the genomics sequence are presented in the Supplementary Table 2. The phylogenetic tree (Figure 5) highlighted the relationship among the samples clustered according to the MPPs and dates of positivity. In total were identified six clusters and four of them (BA.2, BA.1.1, BA.1.19, and BA.1) grouped samples originated from the same MPP. The cluster grouping the lineage BA.1.1.15 included samples from three different MPPs (U, J, and I), while samples from MP R generated two distinct clusters with two lineages (BA.2 and BA.1.19) of the variant Omicron (clade 21L and 21 K respectively).

## Discussion

WvGS can be used to identify VOCs in the population at large (18) and can also be used at higher resolution to support epidemiological investigation of outbreaks (1, 5, 6, 17–19). As more of the Irish population is vaccinated, the application of WvGS becomes increasingly important to quickly identify and control new and emerging variants that could escape vaccinal protection particularly in elderly and vulnerable individuals (6). In this context, the source and spread of future virus outbreaks should be more aggressively tracked and traced to expedite its elimination from the Irish population.

RADTs in which virus has been inactivated have been used for years for example when transferring foot-and-mouth disease virus test samples from remote field locations to reference laboratories for characterization (20). The present study provides proof of the concept that sufficient SARS-CoV-2 viral genome can be readily recovered from positive RADT devices in which the virus has been safely inactivated to allow for high resolution sequencing. This is a useful extended finding which should be viewed in the context outlined above, as providing an additional source of material for WvGS.

Detection of lineages of the Omicron VOC from field samples and one lineage of the Alpha VOC experimentally, means there is no guarantee that other VOCs could be detected by the method described here. Only two different RADTs were used in this study and either the lysis buffers provided or the makeup of the lateral flow devices provided with other RADT kits could prevent the isolation of viral RNA for sequencing by the method described here. Further application of this method to recover RNA from positive RADTs and detect circulating variants will determine if this method can be utilized to detect other VOCs and be used with other RADT kits. Interestingly, the phylogenetic analysis highlighted the relationship among samples, and different clusters were identified, grouping in some cases, samples from the same MPPs. The limited number of samples for this trial and the relatively short period of time of the survey couldn't support more speculations on the direction of the infections however, the significant additional benefit derived from this study was proof of the concept that viral genome sequences could be obtained from spent positive RADTs. As the pandemic has evolved, tracking the spread of VOCs has become a priority for public health authorities. This study demonstrates the possibility of rapidly sequencing viruses associated with infections in workplace environments, such as MPPs, both to monitor the viral variants and lineages in circulation, and in future, with the validation of other available RADTs and the availability of WvGS obtained using this protocol, could be potentially applied to identify sources of infection, and the direction of person-to-person spread within workplaces.

## Data Availability Statement

The datasets presented in this study can be found in online repositories. The names of the repository/repositories and accession number(s) can be found below: https://www.ncbi.nlm.nih.gov/genbank/, ON077632, ON077631, ON075499.1, ON075498.1.

## Author Contributions

SF, DS, and GMu designed the project. GMa, TR, GB, PW, and SF designed the experiments and wrote the manuscript. GMa and TR performed the experiments. GMa performed the genomic analysis. TR and GB cultivated SARS-CoV-2 and performed the experiment in CL3. LK and SM contributed on the extraction and library preparation for sequencing for the field-study. All authors read and approved the submitted version of the manuscript.

## Funding

This work was supported by the COVID Rapid Response Grant 20/COV/8436 from Science Foundation Ireland.

## Conflict of Interest

The authors declare that the research was conducted in the absence of any commercial or financial relationships that could be construed as a potential conflict of interest.

## Publisher's Note

All claims expressed in this article are solely those of the authors and do not necessarily represent those of their affiliated organizations, or those of the publisher, the editors and the reviewers. Any product that may be evaluated in this article, or claim that may be made by its manufacturer, is not guaranteed or endorsed by the publisher.

## Supplementary Material

Supplementary Table 1 Dataset 30 samples of RADTs resulted positive from a survey in MPPs in Ireland. The table presents the qualitative results of the sequencing and report of the results of the Nextstrain SARS-CoV-2 tool.

Supplementary Table 2 Dataset 30 samples of RADTs resulted positive from a survey in MPPs in Ireland. The table presents metadata and results of the RT-qPCR and qualitative results of the sequencing, including Ct values, Nextclade Pango, total Nucleotide Missing and % completion genome.
